# Digital transformation of spiritual care education: A scoping review protocol of technology-enhanced learning for nursing students and professionals

**DOI:** 10.1371/journal.pone.0353851

**Published:** 2026-07-22

**Authors:** Peiyu Sun, Ya Shi, Guo Chen, Yun Chen, Guohua Fan, Yingying Cao

**Affiliations:** 1 School of Nursing, Faculty of Medicine, Yangzhou University, Yangzhou, China; 2 Nursing Research Center, Shandong Xiehe University, Jinan, China; 3 Northern Jiangsu People’s Hospital, Yangzhou, Jiangsu, China; The Hong Kong Polytechnic University, HONG KONG

## Abstract

**Background:**

Spiritual care is a fundamental yet often under-addressed component of holistic nursing, with nurses and nursing students globally reporting limited competency. Traditional education models face challenges in accessibility, scalability, and pedagogical depth. While digital technologies offer transformative potential for learning, the evidence regarding their application and effectiveness in spiritual care education remains fragmented and insufficiently synthesized.

**Objective:**

This scoping review aims to systematically map the existing literature on the use of digital technologies in spiritual care education and training specifically for nurses and nursing students.

**Methods:**

The review will be conducted following the Joanna Briggs Institute (JBI) methodology for scoping reviews and reported per the PRISMA-ScR guidelines. A comprehensive search will be performed across multiple databases (e.g., MEDLINE, CINAHL, PsycINFO, Web of Science, Scopus, CNKI, WanFang) from inception, supplemented by grey literature searches. Two independent reviewers will screen records, select studies, and extract data using a standardized form. We will include empirical studies focusing on digital interventions (e.g., online courses, mobile apps, virtual simulations) designed to develop spiritual care competency in the target population. Data will be analyzed using descriptive statistics and thematic analysis.

**Anticipated results:**

The review will generate a comprehensive map and classification of existing digital spiritual education interventions, detailing their technological characteristics, pedagogical strategies, outcome measures, and implementation facilitators and barriers. It is expected to identify key evidence gaps and inconsistencies in the field. For example, we anticipate identifying virtual simulation, online modules, and mobile apps as the most common categories.

**Significance:**

This will be the first scoping review to systematically consolidate evidence on digital spiritual education for nurses. The findings will provide a foundational resource to inform the future design, implementation, and evaluation of theoretically grounded, effective, and scalable digital training models, ultimately aiming to enhance spiritual care competence in nursing practice.

**Systematic review registration:**

OSF registration number: https://doi.org/10.17605/OSF.IO/7VNHF.

## Introduction

Spiritual care, defined as the aspect of healthcare that addresses the human need for meaning, purpose, and connection in the context of illness and suffering, is a cornerstone of holistic nursing practice [1,2]. Its significance is underscored by the World Health Organization’s recognition of spirituality as a vital component of quality of life [3]. A robust and growing body of evidence demonstrates that patients’ spiritual well-being is positively associated with critical health outcomes, including enhanced coping abilities, improved psychological adjustment, better treatment adherence, and higher overall quality of life [4–6]. For example, a longitudinal study of palliative clients found that structured spiritual care interventions contributed to sustained spiritual well-being over time [5], while a cross‑sectional study among patients with diabetes reported a significant positive association between spiritual wellbeing and treatment adherence [6].Consequently, the ability to competently assess and address spiritual concerns is no longer an optional skill but an ethical and professional imperative for nurses across all clinical settings [7–10].

Despite this clear mandate, a pervasive and well-documented gap exists between the recognized importance of spiritual care and nurses’ readiness to provide it. Global studies consistently report moderate to low levels of spiritual care competence among nursing professionals and students, characterized by deficiencies in knowledge, communication skills, and the confidence to engage in spiritual interventions [11–13]. A recent scoping review and a meta-analysis confirm this troubling trend, highlighting a universal need for effective educational interventions [14,15].

This discrepancy is largely driven by insufficient training, which continues to represent a major barrier to integrating spirituality into routine clinical practice [11,16,17]. Historically, spiritual care education has relied predominantly on conventional, face-to-face formats such as classroom lectures, workshops, and seminars [16,17]. While valuable for foundational knowledge transfer, these models are fraught with systemic limitations that hinder scalability and long-term impact. Key challenges include: (1) Low accessibility and participation: Nurses often face logistical barriers (e.g., shift work, geographical constraints) and low participation rates, sometimes as low as 10%, when training requires time away from clinical duties [11,17,18]; (2) High resource intensity: Traditional programs entail significant costs for instructors, venues, and materials, limiting their sustainability and reach [19,20]; and (3) Pedagogical constraints: These methods often emphasize theoretical content over practical application, offering few opportunities for interactive learning, clinical simulation, or personalized feedback, which are crucial for developing complex relational skills [21–23].

Digital educational technologies, by their very nature, are positioned to address these specific limitations. The digital transformation of healthcare education presents a pivotal opportunity to overcome these barriers. Digital health technologies, encompassing online learning platforms, mobile applications, virtual and augmented reality simulations, and blended learning ecosystems, have revolutionized pedagogical approaches by offering unprecedented flexibility, scalability, and interactivity [24,25]. Several systematic reviews suggest that e‑learning can achieve comparable effects to traditional methods in improving knowledge and skills in nursing education, with potential added benefits in learner engagement and accessibility [24,26,27]. However, the evidence base varies, and findings are not uniformly consistent across all educational contexts or outcome measures. The core strengths of digital interventions, including self-paced learning, just-in-time knowledge access, immersive simulation, and collaborative online communities, align perfectly with the need for continuous, practice-based professional development in spiritual care [28–30].

However, digital education also carries well‑documented risks. Disparities in digital literacy can exacerbate educational inequalities, especially when access to reliable technology or technical support is uneven [24]. The relational depth of face‑to‑face encounters may be diminished in technology‑mediated interactions, raising concerns about the development of empathic communication and therapeutic presence—skills central to spiritual care [31,32]. Engagement and motivation can be difficult to sustain in self‑directed digital formats, particularly when learning demands high levels of reflection or emotional openness [24]. Moreover, digital tools may lack the cultural and contextual sensitivity required to address spiritually nuanced content, as standardised platforms may not easily accommodate diverse religious, philosophical, or personal belief systems. These limitations are especially relevant to spiritual care education, where holistic, person‑centred competence depends heavily on relational practice and contextual understanding.

Nevertheless, the very features, including fixed schedules, geographical constraints, resource intensity, that make traditional spiritual care education inaccessible or unscalable are precisely what digital technologies can mitigate. The key question is not whether digital methods can fully replicate face‑to‑face relational depth, but how they can be designed and deployed to complement existing approaches, reduce barriers to entry, and offer new forms of reflective and simulated practice (e.g., safe repetition of difficult conversations, just‑in‑time knowledge reinforcement). Recognising both potential and perils, a growing number of pioneering studies have begun to explore digital tools specifically for spiritual care education.

Initial implementations range from online courses delivered via platforms like Zoom [33,34] to mobile apps for spiritual assessment training [35] and virtual simulations for practicing difficult conversations [36,37]. Preliminary findings are promising, suggesting positive impacts on learners’ spiritual care knowledge, self-efficacy, attitudes, and perceived competence [35,38,39].

However, this nascent field is characterized by significant fragmentation and methodological heterogeneity. Existing studies vary widely in their technological approaches (e.g., synchronous vs. asynchronous, standalone apps vs. comprehensive platforms), pedagogical designs, intervention durations, and outcome measures. Crucially, there is a conspicuous absence of a scoping review that systematically maps this heterogeneous landscape specifically for nursing populations. A synthesis is needed to consolidate the characteristics of these interventions, their outcome measures, and their implementation challenges to guide future development and research.

Given nurses’ central role as primary providers of continuous, holistic care and the explicit definition of spiritual care as a core nursing competency [40–42], this scoping review will focus specifically on the population of nursing students and registered nurses. Unlike physicians who primarily concentrate on diagnosis and treatment, or chaplains who hold specialized religious roles, nurses serve as round-the-clock bedside caregivers with the most sustained and direct patient contact [43]. Empirical evidence shows that the frequency and quality of nurse-provided spiritual care are directly associated with patient outcomes, including spiritual well-being, treatment adherence, and overall quality of life [6,8,12]. Moreover, the barriers nurses face, including shift work, heavy clinical workloads, and limited access to traditional training, are distinct from those encountered by other spiritual care providers [11,15]. This focused approach ensures depth, coherence, and practical relevance for the nursing profession. The important topic of interprofessional digital spiritual education is thus identified as a distinct and necessary area for future investigation.

The importance of conducting this scoping review lies in its potential to bridge a critical gap at the intersection of two pressing priorities in contemporary nursing education: the urgent need to strengthen spiritual care competencies and the growing reliance on digital learning modalities. Without a comprehensive synthesis of the existing evidence, educators and curriculum developers risk investing in digital interventions that may not align with the pedagogical demands of spiritual care, a domain that requires relational depth, reflective practice, and cultural sensitivity. By systematically mapping the available literature, this review will provide the foundational evidence needed to guide future educational innovation, resource allocation, and research design in this underexplored area.

To address this critical knowledge gap and inform the future development of evidence-based digital education, this scoping review aims to systematically map and synthesize the existing literature on digital technology-assisted spiritual education and training for nurses and nursing students. Specifically, this review seeks to: (1) Identify and categorize the types of digital technologies and pedagogical strategies employed; (2) Synthesize reported outcomes related to spiritual care competence and the tools used to measure them; (3) Explore the facilitators, barriers, and implementation challenges associated with these digital programs; (4) Highlight key research gaps and methodological limitations to guide future inquiry and innovation. A preliminary search of the PROSPERO international prospective register of systematic reviews and the JBI Evidence Synthesis database (conducted in March 2026) did not identify any existing or ongoing scoping reviews on the use of digital technologies in spiritual care education specifically for nurses and nursing students. This confirms that the proposed review will be the first to systematically consolidate evidence in this area.

## Methods

### Review design and framework

This scoping review will be conducted according to the established methodological framework for scoping studies proposed by Arksey and O’Malley [44] and enhanced by the subsequent Joanna Briggs Institute (JBI) guidance [45]. The process will encompass five iterative stages: (1) identifying the research question, (2) identifying relevant studies, (3) study selection, (4) charting the data, and (5) collating, summarizing, and reporting the results. Reporting will adhere to the Preferred Reporting Items for Systematic reviews and Meta-Analyses extension for Scoping Reviews (PRISMA-ScR) checklist [46]. The protocol for this review has been prospectively registered on the Open Science Framework (OSF) (Registration DOI: https://doi.org/10.17605/OSF.IO/7VNHF) to ensure transparency and mitigate reporting bias.

### Stage 1: Identifying the research questions

The review is guided by the following specific research questions (RQs), formulated to address the knowledge gap regarding digital spiritual care education:

(1) What are the characteristics (e.g., types of technology, delivery modes, duration, pedagogical frameworks, and theoretical underpinnings) of digital interventions designed for spiritual care education targeting nursing students and registered nurses?
(2) What outcome measures and assessment tools are utilized to evaluate the impact of these digital interventions on spiritual care competencies, knowledge, attitudes, and behaviors?
(3) What are the reported facilitators, barriers, and challenges associated with the implementation, adoption, and sustainability of these digital education programs?
(4) Based on the synthesized evidence, what key gaps and priorities exist in the current research landscape to inform future study design and educational practice?

### Stage 2: Identifying relevant studies

A systematic and comprehensive search strategy will be developed in consultation with a health sciences librarian. The following electronic databases will be searched from their inception to the present date: PubMed/MEDLINE, Embase, CINAHL Complete, PsycINFO, Web of Science Core Collection, Scopus, the Cochrane Library (including CENTRAL), China National Knowledge Infrastructure (CNKI), WanFang Data, and VIP Database for Chinese Technical Periodicals (VIP). The search strategy will combine controlled vocabulary (e.g., MeSH, Emtree) and free-text keywords related to three core concepts: (1) Population: nurses, nursing students; (2) Intervention: digital technology, e-learning, online education, mobile applications, virtual reality; and (3) Phenomenon of Interest: spiritual care, spiritual education, spirituality. Boolean operators (AND, OR) will be used appropriately. The preliminary search strategy for PubMed is presented in Table 1. This strategy will be adapted for the syntax and functionalities of each database. To minimize publication bias, we will also search for grey literature through Google Scholar, OpenGrey, and relevant professional organization websites (e.g., Sigma Theta Tau International). For grey literature, we will include only documents that provide sufficient methodological detail to assess eligibility (e.g., full conference papers, technical reports, dissertations). For abstracts without full text, we will attempt to contact authors for additional information; if unavailable, the record will be excluded. Furthermore, the reference lists of all included studies and relevant systematic reviews will be hand-searched to identify additional eligible records.

**Table 1:** Preliminary search strategy for MEDLINE.

Set	Query
**#1**	(Nurse Practitioners OR Nursing students OR Faculty, Nursing OR Nursing Staff OR Nursing Care OR Nurse [MeSH Terms]) OR (nurs* OR nursing student OR registered nurse OR nurse practitioner OR clinical nurse OR nurse educator OR nursing staff OR nursing personnel OR nursing care)
**#2**	(Religion and Medicine OR Traditional Medicine Practitioners OR pastoral care [MeSH Terms]) OR (spiritual care OR spiritual support OR spiritual intervention OR spiritual well-being OR religious care OR faith-based OR spiritual need OR spiritual distress OR spiritual health OR spiritual practice OR spiritual coping OR spiritual assessment)
**#3**	(digital health OR Telemedicine OR Education, Distance OR virtual reality OR Mobile Applications OR Smartphone OR Generative Artificial Intelligence OR Artificial Intelligence OR Computer-Assisted Instruction OR social media OR digital technology OR information technology[MeSH Terms]) OR (digital health OR ehealth OR e-health OR mhealth OR m-health OR telehealth OR telemedicine OR online learning OR virtual reality OR mobile app OR mobile application OR smartphone OR wearable OR chatbot OR artificial intelligence OR computer-assisted instruction OR blended learning OR social media OR serious game OR simulation OR digital intervention OR web-based OR internet-based OR digital technology OR information technology)
**#4**	#1 AND #2 AND #3
**Filters**	None (Language, date)

Note: The search strategies for Embase, PsycINFO, Scopus, Web of Science, CNKI, WanFang, and VIP were developed by adapting the MEDLINE strategy. For each database, appropriate controlled vocabulary (e.g., Emtree for Embase) and field codes were used, and free-text terms were adjusted for syntax compatibility.

### Stage 3: Study selection

All retrieved records will be imported into EndNote X20 (Clarivate Analytics) for deduplication. The remaining unique citations will then be uploaded to the web-based systematic review manager Rayyan [47] for the screening process. Study selection will involve two sequential phases: ① Title and Abstract Screening: Two independent reviewers will screen all titles and abstracts against the pre-defined eligibility criteria (Table 2). Articles deemed potentially relevant by either reviewer will proceed to full-text review; ② Full-Text Review: The full texts of all potentially eligible studies will be retrieved and assessed independently by the same two reviewers against the inclusion/exclusion criteria. Discrepancies at either stage will be resolved through discussion between the reviewers. If consensus cannot be reached, a third senior reviewer will be consulted to make a final decision. To assess inter-rater reliability, Cohen’s kappa coefficient will be calculated. Based on established guidelines, such as the Cochrane Handbook [48], a kappa value of ≥0.60 will be considered acceptable, as this threshold indicates “good” to “substantial” agreement. If the kappa value falls below 0.60, reviewers will discuss discrepancies, refine the screening criteria, and repeat screening on a new sample until satisfactory agreement (≥0.60) is achieved [49].

**Table 2:** Eligibility criteria for title and abstract screening.

	Inclusion criteria	Exclusion criteria
**Population**	Studies involving nursing students (pre‑licensure, undergraduate, postgraduate) or registered nurses. Studies with other health professionals are included if separate nurse/nursing student data are available.	Studies focusing only on patients, general public, or non-nursing health professionals without separate nurse data.
**Intervention**	Spiritual care education/training with digital technology as a core component (online courses, MOOCs, mobile apps, VR/AR, virtual simulation, blended learning, webinars).	Digital technology as minor supplement only; purely traditional/non-digital education.
**Outcomes**	Any outcomes related to spiritual care learning or competency (knowledge, attitudes, self‑efficacy, skills, perceived competence, behavioral intention, clinical application).	Only outcomes unrelated to spiritual care learning/competence (e.g., only usability or platform satisfaction).
**Study design**	Empirical studies (RCT, non-RCT, pre-post, cohort, case–control, qualitative); peer-reviewed journal articles, theses/dissertations, conference proceedings with sufficient methodology.	Non-empirical publications: editorials, commentaries, opinions, narrative reviews, case reports, underdetailed abstracts.
**Other**	No restrictions on publication date, language, or region.	Ongoing/unfinished studies; study protocols; full text/data unavailable after ≥2 author contacts.

The study selection process will be documented in detail and presented using a flow diagram adapted from the Preferred Reporting Items for Systematic Reviews and Meta-Analyses extension for Scoping Reviews (PRISMA-ScR) statement [46]. The diagram will illustrate the number of records identified from all sources, duplicates removed, records screened at the title/abstract and full-text levels, and studies included in the final review, with specific reasons for exclusions at the full-text stage. The study selection process is anticipated to be completed within two to three months after the search is conducted, depending on the volume of records retrieved.

### Eligibility criteria

The eligibility criteria for this scoping review were developed using the PICO framework (Population, Intervention, Comparator, Outcomes) [50], supplemented by considerations of study design, language, and publication date. The inclusion criteria are detailed below.

#### Inclusion criteria.

**P-Population:** Studies will be included if they involve nursing students (pre-licensure, undergraduate, or postgraduate) and/or registered nurses in any clinical setting. Studies that include other healthcare professionals or students (e.g., physicians, chaplains) will be included only if they provide separately analyzable data for the nurse/nursing student cohort. Studies focusing exclusively on patients or the general public will be excluded.

**I-Intervention:** We will include educational or training interventions aimed at developing spiritual care competency, where digital technology is a core delivery component. Examples include online courses, MOOCs, mobile applications, virtual simulations, VR/AR, blended learning with significant online modules, and webinars. Interventions in which digital technology serves only as a minor supplementary tool (e.g., email reminders for a face-to-face course) will be excluded, as will purely traditional, non-digital educational methods.

**C-Comparator:** Any comparator will be accepted, including traditional face-to-face teaching, another digital intervention, wait-list control, no intervention, or single-group pre-post designs. No specific comparator is required for inclusion.

**O-Outcomes:** We will include studies reporting any outcome related to spiritual care learning or competency development, such as changes in knowledge, attitudes, self-efficacy, perceived competency, skills, behavioral intention, or clinical application. Studies using validated tools (e.g., Spiritual Care Competence Scale) or qualitative descriptions of outcomes are eligible. Studies that only report outcomes unrelated to learning or competency development (e.g., only usability or satisfaction with the technology platform without assessing spiritual care learning) will be excluded.

**Study design:** Empirical studies will be included, such as randomized controlled trials, non-randomized controlled trials, pre-post studies, cohort studies, case-control studies, and qualitative studies. Peer-reviewed journal articles, published theses/dissertations, and conference proceedings with sufficient methodological detail are eligible.

**Language and publication date:** No restrictions will be placed on publication date or geographical location. No language restrictions will be applied. For studies published in languages other than English or Chinese, translation software (e.g., Google Translate) will be used to assist with screening and data extraction. If the software proves insufficient for accurate interpretation, we will seek assistance from individuals with expertise in the respective language.

#### Exclusion criteria.

(1) Unfinished or ongoing studies, as well as study protocols (including trial registrations without full results), will be excluded.
(2) Studies focusing on populations other than nursing students or registered nurses (e.g., patients, the general public, or healthcare professionals other than nurses) will be excluded, unless they provide separately analyzable data for the target nursing population.
(3) Interventions where digital technology is only a minor supplementary tool (e.g., email reminders for a face-to-face course) or where the educational method is purely traditional and non-digital will be excluded.
(4) Studies that only report outcomes unrelated to spiritual care learning or competency development (e.g., only usability or satisfaction with the technology platform without any assessment of spiritual care knowledge, attitudes, skills, or competency) will be excluded.
(5) Non-empirical publications, including editorials, commentaries, opinion pieces, narrative reviews, conference abstracts without sufficient methodological detail, and case reports, will be excluded.
(6) Studies that otherwise meet the inclusion criteria but for which the full text or necessary outcome data cannot be obtained after contacting the authors (at least two attempts) will be excluded.

### Stage 4: Data extraction

A standardized, pilot-tested data extraction form will be developed based on the JBI template [45]. Two reviewers will independently extract data from each included study. The form will capture the following key information: ① Study Identification: Authors, year, country, publication type, study aims, and research design. ② Participant Characteristics: Sample size, demographics (e.g., role [student/staff], level of experience, clinical area). For studies that include multiple professional groups (e.g., nurses, chaplains, pastoral care workers), data will be extracted separately for nurses and nursing students where available. Studies that focus exclusively on non‑nursing professionals will be excluded unless they provide separate analyzable data for the target population. If a study includes multiple professional groups but does not provide separately analyzable data for nurses or nursing students, it will be excluded from the review and recorded as “excluded – ineligible population” in the PRISMA flow diagram. ③ Intervention Details: A comprehensive description of the digital intervention, including technology type/platform, delivery mode (synchronous/asynchronous), duration/ frequency, core educational content, pedagogical/theoretical framework, and instructional strategies. ④ Outcome Data: Primary and secondary outcomes related to spiritual care competency, measurement tools used (with reliability/validity noted if reported), and key quantitative/qualitative findings. ⑤ Implementation Factors: Reported facilitators, barriers, challenges, and lessons learned related to the development, delivery, or adoption of the intervention. ⑥ Other: Authors’ conclusions and recommendations for future research.

The draft extraction form will be piloted on a random sample of three included studies by both reviewers. Following piloting, the reviewers will meet to discuss discrepancies, clarify coding rules, and refine the form categories for consistency before proceeding with full data extraction. Inter‑rater agreement during data extraction will be assessed by calculating the percentage of agreement on all extracted items for the pilot studies. Any disagreements will be resolved through discussion; if consensus cannot be reached, a third reviewer will be consulted. The refined extraction form and coding rules will then be used for the remaining studies. Data extraction is anticipated to be completed within two to three months after the final study selection, allowing for iterative refinement of the extraction form.

### Stage 5: Data synthesis and presentation

Given the anticipated heterogeneity in study designs and outcomes, a formal meta-analysis is not planned. Instead, we will employ a narrative synthesis approach guided by the research questions. Extracted data will be summarized both descriptively and thematically. ① Descriptive Analysis: Tabular and graphical summaries will present the characteristics of included studies (e.g., publication trends, geographical distribution, study designs). Intervention details will be categorized and compared (e.g., by technology type, pedagogical approach). ② Thematic Analysis: For research questions 2–4, qualitative findings and textual data regarding outcomes, assessment tools, and implementation factors will be analyzed using inductive thematic analysis. Recurrent themes, patterns, and concepts will be identified, categorized, and presented with supporting quotes from the original studies. We will also synthesize the use of theoretical or pedagogical frameworks (e.g., experiential learning theory, cognitive load theory, reflective practice models) across studies, noting whether interventions were theory-driven or atheoretical. For mixed-methods studies, quantitative and qualitative findings will be extracted separately and synthesized alongside findings from purely quantitative or qualitative studies, with attention to convergence or divergence. ③ Gap Analysis: The synthesis will explicitly map the current evidence against the research questions to identify consistencies, contradictions, and most importantly, evidence gaps (e.g., underrepresented populations, unexamined outcomes, lack of long-term follow-up, methodological weaknesses).

Results will be presented in a structured format, integrating summary tables, charts (e.g., bar charts, bubble maps), and narrative summaries to provide a clear, comprehensive, and accessible overview of the field. Descriptive tables will summarize: (a) study characteristics (author, year, country, design); (b) intervention characteristics (technology type, duration, pedagogical approach); (c) outcome measures and key findings. A conceptual diagram (e.g., evidence map) will be developed to visualize the relationships between technology types and educational outcomes. The synthesis process will be iterative, allowing for refinement as the data are collated and analyzed. Data synthesis, analysis, and manuscript preparation are anticipated to be completed within four to six months following data extraction.

### Ethical considerations

As this study involves the synthesis of previously published data, formal ethical approval is not required. However, all findings will be reported with integrity, and original sources will be duly cited.

### Team composition

The research team brings together expertise in nursing education, spiritual care, digital health pedagogy, and systematic review methodology.

## Discussion

This scoping review protocol outlines a systematic approach to map the emerging evidence on digital technology-enabled spiritual education for nurses and nursing students. By synthesizing intervention characteristics, outcome measures, and implementation factors, this review will generate a comprehensive landscape analysis of the field. The anticipated findings are expected to offer significant theoretical, practical, and policy implications for integrating spirituality into nursing education in the digital age.

The primary contribution of this review will be the creation of a structured evidence map that clarifies the current state of knowledge. By categorizing digital interventions (e.g., online modules, virtual simulations, mobile apps) and linking them to pedagogical strategies and outcomes, this work will help identify effective educational components and mechanisms of impact. Theoretically, it may reveal how specific technological affordances (e.g., interactivity, immersion, asynchronous access) support the development of complex spiritual care competencies, such as reflective practice or empathetic communication. For nursing educators and curriculum developers, the synthesis will provide a practical toolkit of evidence-based digital strategies. It will highlight which approaches show promise for improving specific competencies, potentially informing the design of more engaging, scalable, and cost-effective training programs. For healthcare institutions, insights into implementation barriers and facilitators will be crucial for strategic planning and resource allocation to support staff development in spiritual care.

The rigor of this review is underpinned by its adherence to established scoping review methodology (JBI and PRISMA-ScR guidelines) and its comprehensive, inclusive search strategy. The protocol employs a systematic search across multiple interdisciplinary databases (e.g., CINAHL, PsycINFO, MEDLINE) and includes Chinese databases (CNKI, WanFang) and grey literature, minimizing geographical and publication bias. The use of dual, independent reviewers at all stages (screening, extraction) enhances reliability and reduces error. The decision to employ a scoping review methodology is particularly appropriate for this nascent field, as it allows for the broad inclusion of diverse study designs necessary to map the full scope and nature of available evidence, rather than being limited to narrowly defined interventions for a focused efficacy estimate.

Several limitations must be acknowledged. First, the expected heterogeneity in study designs, intervention types, and outcome measures may preclude definitive conclusions about comparative effectiveness and limit quantitative synthesis. Second, the review’s focus on nurses and nursing students, while justified, excludes other vital spiritual care providers (e.g., chaplains, social workers), potentially overlooking valuable interprofessional educational models. Third, as a review of published literature, its conclusions will be constrained by the existing evidence base, which may lack long-term follow-up data, robust controlled trials, and studies from low-resource settings. Fourth, in keeping with the established purpose and methodological guidance for scoping reviews, this study will not include a formal critical appraisal of individual sources. This feature, common to all scoping reviews, means that the synthesis focuses on describing the volume, nature, and scope of the evidence landscape rather than providing an assessment of the quality or certainty of the findings presented. Consequently, our findings will be presented as a descriptive mapping of available interventions, outcomes, and implementation factors, without making claims about the quality or certainty of the evidence (e.g., we will not state that “high‑quality evidence shows” a particular effect). Moreover, it is important to acknowledge that the available literature on digital education interventions may be subject to publication bias, with studies reporting positive findings more likely to be published. This limitation should be considered when interpreting the breadth and nature of the mapped evidence [24].

## Conclusion

This protocol describes a plan to conduct the first comprehensive scoping review dedicated to digital spiritual education in nursing. Despite its limitations, this review is poised to fill a critical knowledge gap by systematically cataloging and analyzing a dispersed body of literature. The resulting evidence map will not only illuminate current practices and their reported outcomes but will also explicitly identify key research priorities. Potential directions for future research may include: 1) developing and evaluating theory-driven digital interventions with explicit pedagogical frameworks; 2) designing rigorous randomized controlled trials with longer-term follow-up to assess sustained competency development; 3) creating and validating technology-sensitive assessment tools for spiritual care skills; and 4) exploring implementation science approaches to scale up successful digital training models across diverse clinical and educational settings. By charting this course, the review aims to catalyze more coordinated, methodologically sound, and impactful research, ultimately fostering the development of spiritual care competence among nurses—supported by appropriate digital solutions—to enable holistic, spiritually sensitive care.

## Supporting information

S1 Checklist PRISMA-ScR checklist. (DOCX)
