# Supplementary material for: Digital transformation of spiritual care education: A scoping review protocol of technology-enhanced learning for nursing students and professionals
Source: PLoS One. 2026 Jul 22;21(7):e0353851. doi: 10.1371/journal.pone.0353851 (PMC13390859; doi:10.1371/journal.pone.0353851)
Supplement: Attachment — Submitted filename: renamed_5bca8.docx [file pone.0353851.s003.docx]

**Response letter**

Dear Editor and reviewer,

Manuscript ID: PONE-D-26-00957

Title: “Digital transformation of spiritual care education: A scoping review protocol of technology-enhanced learning for nursing students and professionals”

We sincerely thank you for handling our manuscript and for your constructive feedback. We also extend our gratitude to the reviewers for their time and insightful comments, which have helped us significantly improve the quality of our work. We have carefully considered all points raised by you and the reviewers and have thoroughly revised the manuscript accordingly. Our point-by-point responses are detailed below.

| **Journal requirements/comments** | **Response** | **Page** |
| --- | --- | --- |
| **Editor:** | | |
| 1. Style requirements – Please ensure the manuscript meets PLOS ONE’s style requirements, including file naming. | We are grateful for this reminder. We have carefully reviewed the PLOS ONE style templates and confirmed that the manuscript follows the required formatting for font, headings, line numbers, page numbers, and reference style. The file naming has been adjusted to meet journal requirements. | Throughout manuscript |
| 2. Title page – Please include the title page within the main manuscript file, not as a separate file. | We appreciate your guidance.We have incorporated the title page into the beginning of the main manuscript file, listing all authors and affiliations as requested. | Page 1-3 |
| 3. Role of funder – Please state what role the funders took in the study, and include this statement in the cover letter. | Thank you for this clarification.We have added the following Role of Funder statement to the cover letter: “The funder (Nursing Research Special Project of Northern Jiangsu Hospital, China, Grant No. YJJ240045) had no role in study design, data collection and analysis, decision to publish, or preparation of the manuscript.” This statement also appears on the title page. | Cover letter; Title page (Page 2) |
| 4. Funding information mismatch – The grant information in ‘Funding Information’ and ‘Financial Disclosure’ sections do not match. | We appreciate your attention to this detail.We have verified that the funding information is now consistent across both sections in the submission system. The correct grant number (YJJ240045) appears in both fields. | Submission system |
| 5. Data Availability Statement – Please provide a complete Data Availability Statement. | Thank you for the reminder. No new data were generated or analysed in support of this work, as this is a scoping review protocol based on previously published literature. All data included in this protocol are available within the manuscript. | Page 2-3 |
| 6. Supporting Information captions – Please include captions at the end of the manuscript and update in‑text citations. | We are grateful for this guidance. We have added a “Supporting Information” section at the end of the manuscript, before the references, with captions for S1 Checklist.PRISMA-ScR checklist.In‑text citations have been updated accordingly. | Page 22 |
| 7. Reviewer‑suggested citations – Please review and evaluate any recommended citations. | Thank you for this advice. We have carefully reviewed the reviewer’s comments and found no explicit recommendation to cite specific previously published works. Nevertheless, we have taken the opportunity to improve the accuracy and completeness of all references (as detailed in response to Reviewer Major Issue 3) and have ensured that all cited works are relevant and correctly support the arguments. | References list (Pages 22–28) |
| Additional Editor Comment – Please ensure the significance of undertaking this scoping review is made more explicit in the introduction/  background. | We sincerely appreciate this suggestion. We added content in the introduction to clarify the review’s significance: no prior/ongoing scoping review on this topic was found in PROSPERO and JBI (March 2026), so this is the first systematic synthesis to consolidate evidence in this area and serve as a foundational resource.This addition effectively addresses your concern regarding the explicit presentation of the review’s significance. Thank you again for your constructive feedback. | Page 10 |
| **Reviewer:** | | |
| Major Issues: |  |  |
| 1. Search Strategy – The Population term “nurs” in Table 1 lacks a wildcard (“nurs*”), which will result in missed records. The strategy does not include MeSH terms alongside free‑text keywords. | We sincerely thank the reviewer for this important methodological observation. We thank the reviewer for this important methodological observation. We have corrected Table 1 by changing “nurs” to “nurs*” to capture variants such as “nurses” and “nursing”. In addition, to improve transparency, we have added a footnote to Table 1 clarifying that this is a simplified example of the free‑text strategy, and that the full search strategy including controlled vocabulary (MeSH/Emtree) is provided as Supporting Information (S3 Search strategy). The full strategy has been uploaded as a supplementary file. | Table 1 (Page 12-13);  S1 Table |
| 2. Pilot Testing and Inter‑Rater Reliability – The authors mention pilot‑testing the extraction form but do not describe how discrepancies were resolved or whether inter‑rater agreement was assessed. | We are grateful for this insightful comment. We have revised the “Study Selection” and “Data Extraction” sections to explicitly describe the process for resolving discrepancies and assessing inter‑rater reliability. Specifically, in the screening phase we now state that Cohen’s kappa will be calculated on a 10% random sample, with a target of ≥0.60; if this threshold is not met, the criteria will be reviewed and screening repeated. For data extraction, we clarify that percentage agreement will be calculated during pilot testing, and any disagreements resolved through discussion or consultation with a third reviewer. | Pages 13-14 |
| 3. Reference Inaccuracies and Misalignments – Several references are incomplete, inaccurate, or misaligned with the argument (Refs 11, 12, 13, 14, 27) | We thank the reviewer for this careful verification. We have thoroughly reviewed and corrected the references as follows:  Original Ref 11 (Ali et al., 2018): Added full publication details: Int J Multidiscip Comp Stud. 2018;5(1-3):27–49.  Original Ref 12 (Taylor et al., 2023): Confirmed this is an international comparative study that supports the claim. No further changes needed.  Original Ref 13 (Harrad et al., 2019): Confirmed this is a review that supports the claim. No further changes needed.  Original Ref 14 (Khalaf et al., 2025): This was a single‑country primary study and has been replaced with a systematic review (Dunning et al., 2025) that appropriately supports the claim of a global trend.  Original Ref 27 (Livesay et al., 2024): Added full bibliographic details: JMIR Med Educ. 2024;10:e54105. doi:10.2196/54105.  Additionally, we have verified all other references for completeness and accuracy. All documents were reviewed, and those that did not meet the journal’s publication requirements were revised. | References list (Pages 22–28) |
| 4. Overstatement in Effectiveness Claims for Digital Education – The statement that digital education is “at least as effective as traditional methods” relies on inconsistent evidence. | We appreciate this important observation. We have revised the wording to be more cautious and evidence‑appropriate. The sentence now reads: “Several systematic reviews suggest that e‑learning can achieve comparable effects to traditional methods in improving knowledge and skills in nursing education, with potential added benefits in learner engagement and accessibility [24,26,27]. However, the evidence base varies, and findings are not uniformly consistent across all educational contexts or outcome measures.” | Page 7 |
| 5. Missing Discussion of Digital Education Limitations – The introduction omits well‑documented risks such as digital literacy divides, reduced relational depth, engagement challenges, and sensitivity to cultural/spiritual nuance. | We are grateful for this constructive suggestion. We have added a new paragraph in the introduction that explicitly acknowledges these limitations. The added text discusses disparities in digital literacy, diminished relational depth in technology‑mediated interactions, engagement challenges, and the difficulty of addressing culturally and spiritually nuanced content through digital platforms. These limitations are linked to the specific context of spiritual care education. | Page 7-8 |
| 6. PRISMA Checklist Misalignment – The protocol includes a PRISMA‑P checklist, but a scoping review requires the PRISMA‑ScR checklist. | Thank you for pointing this out. We have replaced the PRISMA‑P checklist with the appropriate PRISMA‑ScR checklist. The Supporting Information file (S1 Checklist) now contains the PRISMA‑ScR checklist, and the caption has been updated accordingly. The manuscript text correctly references the PRISMA‑ScR guidelines in the methods section. | Supporting Information; Page 22 |
| 7. Ambiguity in the Conclusion Regarding Competency Focus – The conclusion conflates digital competence and spiritual‑care competence. | We appreciate this clarification. We have revised the conclusion to clarify the distinction. The final sentence now reads: “By charting this course, the review aims to catalyze more coordinated, methodologically sound, and impactful research, ultimately fostering the development of spiritual care competence among nurses—supported by appropriate digital solutions—to enable holistic, spiritually sensitive care.” | Page 22 |
| Minor Issues |  |  |
| 1a. Writing Style and Flow – The Introduction contains repetition between paragraphs two and three regarding competency gaps. | We sincerely thank the reviewer for this careful reading. We have restructured the introduction to eliminate repetition. Paragraph 2 now focuses solely on the existence of the competency gap, while paragraph 3 focuses on the limitations of traditional educational approaches as the cause of that gap. The flow is now clearer and avoids redundancy. | Pages 6-7 |
| 1b. Health‑outcome evidence – The authors may wish to broaden the cited evidence base and provide brief descriptions of individual study contributions. | We appreciate this suggestion to strengthen the evidence base. We have added brief descriptions of two recent studies (Liefbroer et al., 2025; Erden Melikoğlu et al., 2025) to illustrate the health‑outcome relevance of spiritual wellbeing. These descriptions are integrated into the existing paragraph without disrupting the narrative flow. | Page 6 |
| 2a. Structure and Clarification – Timeline – The logistic timeline for completing the review (8 months total) may be optimistic. | We are grateful for this realistic observation. We have adjusted the timeline to be more realistic while maintaining the overall scope. Each stage is now described with a flexible time range (e.g., “anticipated to be completed within two to three months”), acknowledging that the actual duration may vary depending on the volume of records retrieved and the complexity of data extraction. | Pages 14，17-19 |
| 2b. Data extraction – chaplaincy/spiritual‑care practitioners – The data extraction plan could be enriched by explicitly noting whether these professionals will be captured. | Thank you for this valuable suggestion. We have added a clarification in the “Participant Characteristics” section of Stage 4. For studies that include multiple professional groups (e.g., nurses, chaplains, pastoral care workers), data will be extracted separately for nurses and nursing students where available. Studies focusing exclusively on non‑nursing professionals will be excluded unless they provide separate analyzable data for the target population. | Page 17-18 |
| 2c. Redundancy – database coverage – Lines 165–167 repeat earlier information about database coverage. | We appreciate this attention to conciseness. We have removed the redundant sentence in Stage 2 that repeated the list of databases. The detailed database list is now presented only once, earlier in the section. | Stage 2 (Page 11-12) |
| 3a. Abstract – “First scoping review” claim – The claim needs support; a brief mention in the Introduction of a preliminary search of PROSPERO and JBI registries would justify the statement. | We are grateful for this suggestion to strengthen the justification. We have added a new paragraph in the introduction (following the description of the review’s focus) that states: “A preliminary search of the PROSPERO international prospective register of systematic reviews and the JBI Evidence Synthesis database (conducted in March 2026) did not identify any existing or ongoing scoping reviews on the use of digital technologies in spiritual care education specifically for nurses and nursing students. This confirms that the proposed review will be the first to systematically consolidate evidence in this area.” The abstract statement remains unchanged as it is now supported by the text. | Pages 10 |
| 3b. Abstract – pilot‑testing description – The abstract refers to pilot‑testing without describing the procedure. | Thank you for this observation. We have simplified the abstract by replacing “using a pilot‑tested form” with “using a standardized form,” as the detailed pilot‑testing procedure is fully described in the Methods section (Stage 4). | Abstract, Page 4-5 |
| 4. Limitations Section – Publication bias – The manuscript could acknowledge the likelihood of publication bias in digital‑education interventions. | We appreciate this important addition. We have added a brief “Team composition” section after “Ethical Considerations,” stating: “The research team brings together expertise in nursing education, spiritual care, digital health pedagogy, and systematic review methodology.” This highlights the multidisciplinary nature of the team and its relevance to the topic. | Page 21 |
| 5. Team Composition – Explaining the interdisciplinary background of the research team would strengthen credibility. | We are grateful for this suggestion. We have added a brief “Team composition” section after “Ethical Considerations,” stating: “The research team brings together expertise in nursing education, spiritual care, digital health pedagogy, and systematic review methodology.” This highlights the multidisciplinary nature of the team and its relevance to the topic. | Page 19 |

Finally, we really appreciate all the time and effort you put into this document, as well as all your insightful suggestions for the revisions of this manuscript to make it more readable and clearer. Thank you so much once more!

Best wishes
