# Supplementary material for: Digital transformation of spiritual care education: A scoping review protocol of technology-enhanced learning for nursing students and professionals
Source: PLoS One. 2026 Jul 22;21(7):e0353851. doi: 10.1371/journal.pone.0353851 (PMC13390859; doi:10.1371/journal.pone.0353851)
Supplement: Peer review history

# Peer review: Decision Letter 0

**DOI:** 10.1371/journal.pone.0353851.r001

**Authors:** JONATHAN BAYUO

23 Mar 2026

PONE-D-26-00957

Digital transformation of spiritual care education:A scoping review protocol of technology-enhanced learning for nursing students and professionals

PLOS One

Dear Dr. Cao,

Thank you for submitting your manuscript to PLOS ONE. After careful consideration, we feel that it has merit but does not fully meet PLOS ONE’s publication criteria as it currently stands. Therefore, we invite you to submit a revised version of the manuscript that addresses the points raised during the review process.

Please submit your revised manuscript by May 07 2026 11:59PM. If you will need more time than this to complete your revisions, please reply to this message or contact the journal office at plosone@plos.org. When you’re ready to submit your revision, log on to https://www.editorialmanager.com/pone/ and select the 'Submissions Needing Revision' folder to locate your manuscript file.

Please include the following items when submitting your revised manuscript:

- A letter that responds to each point raised by the academic editor and reviewer(s). You should upload this letter as a separate file labeled 'Response to Reviewers'.
- A marked-up copy of your manuscript that highlights changes made to the original version. You should upload this as a separate file labeled 'Revised Manuscript with Track Changes'.
- An unmarked version of your revised paper without tracked changes. You should upload this as a separate file labeled 'Manuscript'.

If you would like to make changes to your financial disclosure, please include your updated statement in your cover letter. Guidelines for resubmitting your figure files are available below the reviewer comments at the end of this letter.

If applicable, we recommend that you deposit your laboratory protocols in protocols.io to enhance the reproducibility of your results. Protocols.io assigns your protocol its own identifier (DOI) so that it can be cited independently in the future. For instructions see: https://journals.plos.org/plosone/s/submission-guidelines#loc-laboratory-protocols. Additionally, PLOS ONE offers an option for publishing peer-reviewed Lab Protocol articles, which describe protocols hosted on protocols.io. Read more information on sharing protocols at https://plos.org/protocols?utm_medium=editorial-email&utm_source=authorletters&utm_campaign=protocols.

We look forward to receiving your revised manuscript.

Kind regards,

JONATHAN BAYUO, PhD

Academic Editor

PLOS One

Journal Requirements:

When submitting your revision, we need you to address these additional requirements.

1. Please ensure that your manuscript meets PLOS ONE's style requirements, including those for file naming. The PLOS ONE style templates can be found at

https://journals.plos.org/plosone/s/file?id=wjVg/PLOSOne_formatting_sample_main_body.pdf and

https://journals.plos.org/plosone/s/file?id=ba62/PLOSOne_formatting_sample_title_authors_affiliations.pdf

2. Please ensure that you include a title page within your main document. We do appreciate that you have a title page document uploaded as a separate file, however, as per our author guidelines (http://journals.plos.org/plosone/s/submission-guidelines#loc-title-page) we do require this to be part of the manuscript file itself and not uploaded separately.

Could you therefore please include the title page into the beginning of your manuscript file itself, listing all authors and affiliations

3. Thank you for stating the following financial disclosure:

"This work was supported by the Nursing Research Special Project of Jiangsu Province's Northern Jiangsu Hospital, China (Grant No. YJJ240045 to Yingying Cao)."

Please state what role the funders took in the study.  If the funders had no role, please state: "The funders had no role in study design, data collection and analysis, decision to publish, or preparation of the manuscript."

If this statement is not correct you must amend it as needed.

Please include this amended Role of Funder statement in your cover letter; we will change the online submission form on your behalf.

4. We note that the grant information you provided in the ‘Funding Information’ and ‘Financial Disclosure’ sections do not match.

When you resubmit, please ensure that you provide the correct grant numbers for the awards you received for your study in the ‘Funding Information’ section

5. Please provide a complete Data Availability Statement in the submission form, ensuring you include all necessary access information or a reason for why you are unable to make your data freely accessible. If your research concerns only data provided within your submission, please write "All data are in the manuscript and/or supporting information files" as your Data Availability Statement.

6. Please include captions for your Supporting Information files at the end of your manuscript, and update any in-text citations to match accordingly. Please see our Supporting Information guidelines for more information: http://journals.plos.org/plosone/s/supporting-information.

7. If the reviewer comments include a recommendation to cite specific previously published works, please review and evaluate these publications to determine whether they are relevant and should be cited. There is no requirement to cite these works unless the editor has indicated otherwise.

Additional Editor Comments:

In addition to the comments raised by the reviewer, kindly ensure the significance of undertaking this scoping review is made more explicit in the introduction/ background.

[Note: HTML markup is below. Please do not edit.]

Reviewers' comments:

Reviewer's Responses to Questions

**Comments to the Author**

1. Does the manuscript provide a valid rationale for the proposed study, with clearly identified and justified research questions?

The research question outlined is expected to address a valid academic problem or topic and contribute to the base of knowledge in the field.

Reviewer #1: Yes

**********

2. Is the protocol technically sound and planned in a manner that will lead to a meaningful outcome and allow testing the stated hypotheses?

The manuscript should describe the methods in sufficient detail to prevent undisclosed flexibility in the experimental procedure or analysis pipeline, including sufficient outcome-neutral conditions (e.g. necessary controls, absence of floor or ceiling effects) to test the proposed hypotheses and a statistical power analysis where applicable. As there may be aspects of the methodology and analysis which can only be refined once the work is undertaken, authors should outline potential assumptions and explicitly describe what aspects of the proposed analyses, if any, are exploratory.

Reviewer #1: Yes

**********

3. Is the methodology feasible and described in sufficient detail to allow the work to be replicable?

Descriptions of methods and materials in the protocol should be reported in sufficient detail for another researcher to reproduce all experiments and analyses. The protocol should describe the appropriate controls, sample size calculations, and replication needed to ensure that the data are robust and reproducible.

Reviewer #1: No

**********

4. Have the authors described where all data underlying the findings will be made available when the study is complete?

The PLOS Data policy requires authors to make all data underlying the findings described in their manuscript fully available without restriction, with rare exception, at the time of publication. The data should be provided as part of the manuscript or its supporting information, or deposited to a public repository. For example, in addition to summary statistics, the data points behind means, medians and variance measures should be available. If there are restrictions on publicly sharing data—e.g. participant privacy or use of data from a third party—those must be specified.

Reviewer #1: Yes

**********

5. Is the manuscript presented in an intelligible fashion and written in standard English?

PLOS ONE does not copyedit accepted manuscripts, so the language in submitted articles must be clear, correct, and unambiguous. Any typographical or grammatical errors should be corrected at revision, so please note any specific errors here.

Reviewer #1: Yes

**********

6. Review Comments to the Author

Please use the space provided to explain your answers to the questions above and, if applicable, provide comments about issues authors must address before this protocol can be accepted for publication. You may also include additional comments for the author, including concerns about research or publication ethics.

You may also provide optional suggestions and comments to authors that they might find helpful in planning their study.

(Please upload your review as an attachment if it exceeds 20,000 characters)

Reviewer #1: PLOS ONE Peer Review Report

Manuscript: PONE-D-26-00957

Title: Digital transformation of spiritual care education: A scoping review protocol of technology-enhanced learning for nursing students and professionals

Article type: Study Protocol

Dear authors,

Thank you for submitting this manuscript. I read it with genuine interest and appreciation. Spiritual care remains one of the most underserved areas in nursing education research, and the attempt to systematically map the role of digital technologies in this space is both timely and commendable. The authors have clearly invested effort in grounding this protocol within established methodological frameworks.

The research team is to be congratulated for taking on a topic that sits at the intersection of several complex fields — digital health education, professional nursing practice, and spiritual wellbeing — and for doing so with a structured, transparent approach.

The comments that follow are intended in a constructive spirit. They aim to strengthen what is already a solid foundation, and I hope the authors will find them useful as they move this important work forward.

1. Summary of the Research and Overall Assessment

In this protocol, the authors present a scoping review that aims to map the state of digital and technology‑enhanced learning in spiritual care education for nurses and nursing students. The topic is timely, as spiritual care remains an underdeveloped competency in nursing curricula, and the integration of digital education approaches in this domain has not yet been systematically explored. The protocol is grounded in established methodological frameworks (JBI + PRISMA‑ScR), and the authors articulate a clear rationale for examining a field at the intersection of nursing education, digital pedagogy, and spiritual care.

The manuscript is generally well structured and provides a solid rationale. Nevertheless, several methodological, structural, and stylistic issues must be addressed to ensure transparency and reproducibility. Clarifications are needed regarding the search strategy, inter‑rater reliability procedures, the accuracy and completeness of references, and the alignment with PRISMA‑ScR guidelines. Strengthening these areas will enhance both the rigor and clarity of the protocol.

2. Major Issues (must be addressed)

1. Search Strategy:

The Population term “nurs” in Table 1 lacks a wildcard (“nurs*”), which will result in missed records. This risks missing common variants such as nurses, nursing, and nursed. The authors should verify that all truncation wildcards are correctly applied across all sets and databases.

More importantly, the strategy as presented does not include MeSH terms alongside free-text keywords, despite the methods section explicitly stating that “controlled vocabulary (e.g., MeSH, Emtree) and free-text keywords” will be combined. The table could be expanded to reflect both, or at minimum the authors should explain that the table is intentionally a simplified illustration and that the full strategy will be made available as supplementary material.

2. Pilot Testing and Inter‑Rater Reliability Not Fully Described

The authors mention pilot‑testing the extraction form on three studies but do not describe:

- how discrepancies were resolved

- whether inter‑rater agreement was assessed

A clear procedure for screening and extraction reliability is essential for protocol transparency.

3. Reference Inaccuracies and Misalignments

Several references cited to support key claims are incomplete, inaccurate, or misaligned with the argument:

- Reference 11 lacks publication details.

- References 12 and 13 should be checked to ensure they support the claim about spiritual care competency at an international level.

- Reference 14 is cited as a systematic review/meta‑analysis but appears to be a single‑country primary study; the citation should be corrected.

- Reference 27 also lacks bibliographic information.

These issues must be resolved for the scientific accuracy of the introduction.

4. Overstatement in Effectiveness Claims for Digital Education

The statement that digital education is “at least as effective as traditional methods” relies on inconsistent evidence (including older studies and a preprint). This should either be updated with stronger evidence or more cautiously worded.

5. Missing Discussion of Digital Education Limitations

The introduction presents an optimistic rationale but omits well‑documented risks (e.g., digital literacy divides, reduced relational depth, engagement challenges, sensitivity to cultural/spiritual nuance). A brief acknowledgment would result in a more balanced justification for the review.

6. PRISMA Checklist Misalignment

The protocol includes a PRISMA‑P checklist. Because this is a scoping review, the appropriate standard is the PRISMA‑ScR checklist.

7. Ambiguity in the Conclusion Regarding Competency Focus

The conclusion conflates digital competence and spiritual‑care competence. These are distinct; the authors should clarify which is central to the review’s aims and contextualize how the two relate.

3. Minor Issues

1. Writing Style and Flow

The Introduction contains repetition between paragraphs two and three regarding competency gaps; these could be tightened for narrative flow.

When citing the health‑outcome relevance of spiritual well‑being, the authors may wish to broaden the cited evidence base and provide brief descriptions of individual study contributions.

2. Structure and Clarification

The logistic timeline for completing the review (8 months total) may be optimistic given the volume of databases and grey literature. Authors may wish to justify or adjust the timeline.

The data extraction plan could be enriched by explicitly noting whether chaplaincy or spiritual‑care practitioners will be captured or coded within the data extraction framework. Given the interdisciplinary nature of spiritual care, this would add valuable dimension.

Lines 165–167 repeat earlier information about database coverage and should be integrated to avoid redundancy.

3. Abstract

The claim that “This will be the first scoping review…” needs support. A brief mention in the Introduction of a preliminary search of PROSPERO and JBI registries would justify the statement.

The abstract refers to pilot‑testing the data extraction form without describing the procedure. Either remove or briefly clarify.

4. Limitations Section

The manuscript could acknowledge the likelihood of publication bias in digital‑education interventions, even if risk‑of‑bias assessment is not part of a scoping review.

5. Team Composition

Although optional, explaining the interdisciplinary background of the research team (e.g., involving chaplaincy, educational design) would strengthen credibility given the topic's multidimensional nature.

**********

7. PLOS authors have the option to publish the peer review history of their article (what does this mean?). If published, this will include your full peer review and any attached files.

If you choose “no”, your identity will remain anonymous but your review may still be made public.

**Do you want your identity to be public for this peer review?**  For information about this choice, including consent withdrawal, please see our Privacy Policy.

Reviewer #1: **Yes:** Niecky Fruneaux - van Amerongen

**********

[NOTE: If reviewer comments were submitted as an attachment file, they will be attached to this email and accessible via the submission site. Please log into your account, locate the manuscript record, and check for the action link "View Attachments". If this link does not appear, there are no attachment files.]

To ensure your figures meet our technical requirements, please review our figure guidelines: https://journals.plos.org/plosone/s/figures

You may also use PLOS’s free figure tool, NAAS, to help you prepare publication quality figures: https://journals.plos.org/plosone/s/figures#loc-tools-for-figure-preparation.

NAAS will assess whether your figures meet our technical requirements by comparing each figure against our figure specifications.

# Author comment: Author response to Decision Letter 1

**DOI:** 10.1371/journal.pone.0353851.r002

26 May 2026

Dear Editor and reviewer,

We sincerely thank you for handling our manuscript and for your constructive feedback. We also extend our gratitude to the reviewers for their time and insightful comments, which have helped us significantly improve the quality of our work. We have carefully considered all points raised by you and the reviewers and have thoroughly revised the manuscript accordingly. Our point-by-point responses are detailed below.

Response to Editor

Editor Comment 1: Style requirements -- Please ensure the manuscript meets PLOS ONE's style requirements, including file naming.

Response: We are grateful for this reminder. We have carefully reviewed the PLOS ONE style templates and confirmed that the manuscript follows the required formatting for font, headings, line numbers, page numbers, and reference style. The file naming has been adjusted to meet journal requirements.

(Change appears throughout the manuscript)

Editor Comment 2: Title page -- Please include the title page within the main manuscript file, not as a separate file.

Response: We appreciate your guidance. We have incorporated the title page into the beginning of the main manuscript file, listing all authors and affiliations as requested.

(Change appears on Pages 1-3)

Editor Comment 3: Role of funder -- Please state what role the funders took in the study, and include this statement in the cover letter.

Response: Thank you for this clarification. We have added the following Role of Funder statement to the cover letter: "The funder (Nursing Research Special Project of Northern Jiangsu Hospital, China, Grant No. YJJ240045) had no role in study design, data collection and analysis, decision to publish, or preparation of the manuscript." This statement also appears on the title page.

(Change appears in Cover letter; Title page, Page 2)

Editor Comment 4: Funding information mismatch -- The grant information in 'Funding Information' and 'Financial Disclosure' sections do not match.

Response: We appreciate your attention to this detail. We have verified that the funding information is now consistent across both sections in the submission system. The correct grant number (YJJ240045) appears in both fields.

(Change appears in the submission system)

Editor Comment 5: Data Availability Statement -- Please provide a complete Data Availability Statement.

Response: Thank you for the reminder. Thank you for the reminder. No new data were generated or analysed in support of this work, as this is a scoping review protocol based on previously published literature. All data included in this protocol are available within the manuscript.

(Change appears on Pages 2-3)

Editor Comment 6: Supporting Information captions -- Please include captions at the end of the manuscript and update in‑text citations.

Response: We are grateful for this guidance. We have added a "Supporting Information" section at the end of the manuscript, before the references, with captions for S1 Checklist. PRISMA-ScR checklist. In‑text citations have been updated accordingly.

(Change appears on Page 22)

Editor Comment 7: Reviewer‑suggested citations -- Please review and evaluate any recommended citations.

Response: Thank you for this advice. We have carefully reviewed the reviewer's comments and found no explicit recommendation to cite specific previously published works. Nevertheless, we have taken the opportunity to improve the accuracy and completeness of all references (as detailed in response to Reviewer Major Issue 3) and have ensured that all cited works are relevant and correctly support the arguments.

(Change appears on Pages 22–28)

Additional Editor Comment: Please ensure the significance of undertaking this scoping review is made more explicit in the introduction/background.

Response: We sincerely appreciate this suggestion. We added content in the introduction to clarify the review's significance: no prior/ongoing scoping review on this topic was found in PROSPERO and JBI (March 2026), so this is the first systematic synthesis to consolidate evidence in this area and serve as a foundational resource. This addition effectively addresses your concern. Thank you again for your constructive feedback.

(Change appears on Page 10)

Response to Reviewer

Major Issue 1: Search Strategy -- The Population term "nurs" in Table 1 lacks a wildcard ("nurs*"), which will result in missed records. The strategy does not include MeSH terms alongside free‑text keywords.

Response: We sincerely thank the reviewer for this important methodological observation. We have corrected Table 1 by changing "nurs" to "nurs*". In addition, to improve transparency, we have added a footnote to Table 1 clarifying that this is a simplified example of the free‑text strategy, and that the full search strategy including controlled vocabulary (MeSH/Emtree) is provided as Supporting Information (S1 Search strategy). The full strategy has been uploaded as a supplementary file.

(Change appears in Table 1, Pages 12-13)

Major Issue 2: Pilot Testing and Inter‑Rater Reliability -- The authors mention pilot‑testing the extraction form but do not describe how discrepancies were resolved or whether inter‑rater agreement was assessed.

Response: We are grateful for this insightful comment. We have revised the "Study Selection" and "Data Extraction" sections to explicitly describe the process for resolving discrepancies and assessing inter‑rater reliability. Specifically, in the screening phase we now state that Cohen's kappa will be calculated on a 10% random sample, with a target of ≥0.60; if this threshold is not met, the criteria will be reviewed and screening repeated. For data extraction, we clarify that percentage agreement will be calculated during pilot testing, and any disagreements resolved through discussion or consultation with a third reviewer.

(Change appears on Page 13)

Major Issue 3: Reference Inaccuracies and Misalignments -- Several references are incomplete, inaccurate, or misaligned with the argument (Refs 11, 12, 13, 14, 27).

Response: We thank the reviewer for this careful verification. We have thoroughly reviewed and corrected the references as follows:

- Original Ref 11 (Ali et al., 2018): Added full publication details: Int J Multidiscip Comp Stud. 2018;5(1-3):27-49.

- Original Ref 12 (Taylor et al., 2023): Confirmed this is an international comparative study that supports the claim. No further changes needed.

- Original Ref 13 (Harrad et al., 2019): Confirmed this is a review that supports the claim. No further changes needed.

- Original Ref 14 (Khalaf et al., 2025): This was a single‑country primary study and has been replaced with a systematic review (Dunning et al., 2025) that appropriately supports the claim of a global trend.

- Original Ref 27 (Livesay et al., 2024): Added full bibliographic details: JMIR Med Educ. 2024;10:e54105. doi:10.2196/54105.

Additionally, we have verified all other references for completeness and accuracy. All documents were reviewed, and those that did not meet the journal's publication requirements were revised.

(Change appears in References list, Pages 22-28)

Major Issue 4: Overstatement in Effectiveness Claims for Digital Education -- The statement that digital education is "at least as effective as traditional methods" relies on inconsistent evidence.

Response: We appreciate this important observation. We have revised the wording to be more cautious and evidence‑appropriate. The sentence now reads: "Several systematic reviews suggest that e‑learning can achieve comparable effects to traditional methods in improving knowledge and skills in nursing education, with potential added benefits in learner engagement and accessibility [24,26,27]. However, the evidence base varies, and findings are not uniformly consistent across all educational contexts or outcome measures."

(Change appears on Page 7)

Major Issue 5: Missing Discussion of Digital Education Limitations -- The introduction omits well‑documented risks such as digital literacy divides, reduced relational depth, engagement challenges, and sensitivity to cultural/spiritual nuance.

Response: We are grateful for this constructive suggestion. We have added a new paragraph in the introduction that explicitly acknowledges these limitations. The added text discusses disparities in digital literacy, diminished relational depth in technology‑mediated interactions, engagement challenges, and the difficulty of addressing culturally and spiritually nuanced content through digital platforms. These limitations are linked to the specific context of spiritual care education.

(Change appears on Pages 7-8)

Major Issue 6: PRISMA Checklist Misalignment -- The protocol includes a PRISMA‑P checklist, but a scoping review requires the PRISMA‑ScR checklist.

Response: Thank you for pointing this out. We have replaced the PRISMA‑P checklist with the appropriate PRISMA‑ScR checklist. The Supporting Information file (S1 Checklist) now contains the PRISMA‑ScR checklist, and the caption has been updated accordingly. The manuscript text correctly references the PRISMA‑ScR guidelines in the methods section.

(Change appears in Supporting Information; Page 22)

Major Issue 7: Ambiguity in the Conclusion Regarding Competency Focus -- The conclusion conflates digital competence and spiritual‑care competence.

Response: We appreciate this clarification. We have revised the conclusion to clarify the distinction. The final sentence now reads: "By charting this course, the review aims to catalyze more coordinated, methodologically sound, and impactful research, ultimately fostering the development of spiritual care competence among nurses---supported by appropriate digital solutions---to enable holistic, spiritually sensitive care."

(Change appears on Page 22)

Minor Issue 1a: Writing Style and Flow -- The Introduction contains repetition between paragraphs two and three regarding competency gaps.

Response: We sincerely thank the reviewer for this careful reading. We have restructured the introduction to eliminate repetition. Paragraph 2 now focuses solely on the existence of the competency gap, while paragraph 3 focuses on the limitations of traditional educational approaches as the cause of that gap. The flow is now clearer and avoids redundancy.

(Change appears on Pages 6-7)

Minor Issue 1b: Health‑outcome evidence -- The authors may wish to broaden the cited evidence base and provide brief descriptions of individual study contributions.

Response: We appreciate this suggestion to strengthen the evidence base. We have added brief descriptions of two recent studies (Liefbroer et al., 2025; Erden Melikoğlu et al., 2025) to illustrate the health‑outcome relevance of spiritual wellbeing. These descriptions are integrated into the existing paragraph without disrupting the narrative flow.

(Change appears on Page 6)

Minor Issue 2a: Timeline -- The logistic timeline for completing the review (8 months total) may be optimistic.

Response: We are grateful for this realistic observation. We have adjusted the timeline to be more realistic while maintaining the overall scope. Each stage is now described with a flexible time range (e.g., "anticipated to be completed within two to three months"), acknowledging that the actual duration may vary depending on the volume of records retrieved and the complexity of data extraction.

(Change appears on Pages 14, 17-19)

Minor Issue 2b: Data extraction -- chaplaincy/spiritual‑care practitioners -- The data extraction plan could be enriched by explicitly noting whether these professionals will be captured.

Response: Thank you for this valuable suggestion. We have added a clarification in the "Participant Characteristics" section of Stage 4. For studies that include multiple professional groups (e.g., nurses, chaplains, pastoral care workers), data will be extracted separately for nurses and nursing students where available. Studies focusing exclusively on non‑nursing professionals will be excluded unless they provide separate analyzable data for the target population.

(Change appears on Pages 17-18)

Minor Issue 2c: Redundancy -- database coverage -- Lines 165-167 repeat earlier information about database coverage.

Response: We appreciate this attention to conciseness. We have removed the redundant sentence in Stage 2 that repeated the list of databases. The detailed database list is now presented only once, earlier in the section.

(Change appears in Stage 2, Pages 11-12)

Minor Issue 3a: Abstract -- "First scoping review" claim -- The claim needs support; a brief mention in the Introduction of a preliminary search of PROSPERO and JBI registries would justify the statement.

Response: We are grateful for this suggestion to strengthen the justification. We have added a new paragraph in the introduction (following the description of the review's focus) that states: "A preliminary search of the PROSPERO international prospective register of systematic reviews and the JBI Evidence Synthesis database (conducted in March 2026) did not identify any existing or ongoing scoping reviews on the use of digital technologies in spiritual care education specifically for nurses and nursing students. This confirms that the proposed review will be the first to systematically consolidate evidence in this area." The abstract statement remains unchanged as it is now supported by the text.

(Change appears on Page 10)

Minor Issue 3b: Abstract -- pilot‑testing description -- The abstract refers to pilot‑testing without describing the procedure.

Response: Thank you for this observation. We have simplified the abstract by replacing "using a pilot‑tested form" with "using a standardized form," as the detailed pilot‑testing procedure is fully described in the Methods section (Stage 4).

(Change appears in Abstract, Pages 4-5)

Minor Issue 4: Limitations Section -- Publication bias -- The manuscript could acknowledge the likelihood of publication bias in digital‑education interventions.

Response: We appreciate this important addition. We have added a brief paragraph in the limitations section that acknowledges the potential for publication bias, as studies with positive results regarding digital education interventions are more likely to be published. This limitation and its potential impact on the review's findings are now discussed.

(Change appears on Page 21)

Minor Issue 5: Team Composition -- Explaining the interdisciplinary background of the research team would strengthen credibility.

Response: We are grateful for this suggestion. We have added a brief "Team composition" section after "Ethical Considerations," stating: "The research team brings together expertise in nursing education, spiritual care, digital health pedagogy, and systematic review methodology." This highlights the multidisciplinary nature of the team and its relevance to the topic.

(Change appears on Page 19)

Finally, we really appreciate all the time and effort you put into this document, as well as all your insightful suggestions for the revisions of this manuscript to make it more readable and clearer. Thank you so much once more!

Best wishes,

The Corresponding Author

Attachment Submitted filename: renamed_5bca8.docx

# Peer review: Decision Letter 1

**DOI:** 10.1371/journal.pone.0353851.r003

**Authors:** JONATHAN BAYUO

30 Jun 2026

Digital transformation of spiritual care education:A scoping review protocol of technology-enhanced learning for nursing students and professionals

PONE-D-26-00957R1

Dear Dr. Cao,

We’re pleased to inform you that your manuscript has been judged scientifically suitable for publication and will be formally accepted for publication once it meets all outstanding technical requirements.

Within one week, you’ll receive an e-mail detailing the required amendments. When these have been addressed, you’ll receive a formal acceptance letter and your manuscript will be scheduled for publication.

An invoice will be generated when your article is formally accepted. Please note, if your institution has a publishing partnership with PLOS and your article meets the relevant criteria, all or part of your publication costs will be covered. Please make sure your user information is up-to-date by logging into Editorial Manager at Editorial Manager® and clicking the ‘Update My Information' link at the top of the page. For questions related to billing, please contact billing support.

If your institution or institutions have a press office, please notify them about your upcoming paper to help maximize its impact. If they’ll be preparing press materials, please inform our press team as soon as possible -- no later than 48 hours after receiving the formal acceptance. Your manuscript will remain under strict press embargo until 2 pm Eastern Time on the date of publication. For more information, please contact onepress@plos.org.

Kind regards,

JONATHAN BAYUO, PhD

Academic Editor

PLOS One

Additional Editor Comments (optional):

Reviewers' comments:

Reviewer's Responses to Questions

**Comments to the Author**

1. Does the manuscript provide a valid rationale for the proposed study, with clearly identified and justified research questions?

The research question outlined is expected to address a valid academic problem or topic and contribute to the base of knowledge in the field.

Reviewer #1: Yes

**********

2. Is the protocol technically sound and planned in a manner that will lead to a meaningful outcome and allow testing the stated hypotheses?

The manuscript should describe the methods in sufficient detail to prevent undisclosed flexibility in the experimental procedure or analysis pipeline, including sufficient outcome-neutral conditions (e.g. necessary controls, absence of floor or ceiling effects) to test the proposed hypotheses and a statistical power analysis where applicable. As there may be aspects of the methodology and analysis which can only be refined once the work is undertaken, authors should outline potential assumptions and explicitly describe what aspects of the proposed analyses, if any, are exploratory.

Reviewer #1: Yes

**********

3. Is the methodology feasible and described in sufficient detail to allow the work to be replicable?

Descriptions of methods and materials in the protocol should be reported in sufficient detail for another researcher to reproduce all experiments and analyses. The protocol should describe the appropriate controls, sample size calculations, and replication needed to ensure that the data are robust and reproducible.

Reviewer #1: Yes

**********

4. Have the authors described where all data underlying the findings will be made available when the study is complete?

The PLOS Data policy requires authors to make all data underlying the findings described in their manuscript fully available without restriction, with rare exception, at the time of publication. The data should be provided as part of the manuscript or its supporting information, or deposited to a public repository. For example, in addition to summary statistics, the data points behind means, medians and variance measures should be available. If there are restrictions on publicly sharing data—e.g. participant privacy or use of data from a third party—those must be specified.

Reviewer #1: Yes

**********

5. Is the manuscript presented in an intelligible fashion and written in standard English?

PLOS ONE does not copyedit accepted manuscripts, so the language in submitted articles must be clear, correct, and unambiguous. Any typographical or grammatical errors should be corrected at revision, so please note any specific errors here.

Reviewer #1: Yes

**********

6. Review Comments to the Author

Please use the space provided to explain your answers to the questions above and, if applicable, provide comments about issues authors must address before this protocol can be accepted for publication. You may also include additional comments for the author, including concerns about research or publication ethics.

You may also provide optional suggestions and comments to authors that they might find helpful in planning their study.

(Please upload your review as an attachment if it exceeds 20,000 characters)

Reviewer #1: Dear Authors,

Thank you for the opportunity to review your manuscript titled “Digital Transformation of Spiritual Care Education: A Scoping Review Protocol of Technology-Enhanced Learning for Nursing Students and Professionals.”

I would like to express my appreciation for the careful and thorough way in which you have addressed the comments raised in the previous review. In my opinion, the revisions have significantly strengthened the clarity and quality of the manuscript.

I find the topic highly relevant and timely, particularly given the increasing importance of (digital innovation in) nursing education and spiritual care. I am confident that this scoping review will provide valuable insights into the current state of technology-enhanced learning in this field.

I look forward with great interest to the outcomes of your study and would like to wish you every success in its execution.

Kind regards.

**********

7. PLOS authors have the option to publish the peer review history of their article (what does this mean?). If published, this will include your full peer review and any attached files.

If you choose “no”, your identity will remain anonymous but your review may still be made public.

**Do you want your identity to be public for this peer review?**  For information about this choice, including consent withdrawal, please see our Privacy Policy.

Reviewer #1: No

**********

# Editor report: Acceptance letter

**DOI:** 10.1371/journal.pone.0353851.r004

**Authors:** JONATHAN BAYUO

PONE-D-26-00957R1

PLOS One

Dear Dr. Cao,

I'm pleased to inform you that your manuscript has been deemed suitable for publication in PLOS One. Congratulations! Your manuscript is now being handed over to our production team.

At this stage, our production department will prepare your paper for publication. This includes ensuring the following:

* All references, tables, and figures are properly cited

* All relevant supporting information is included in the manuscript submission,

* There are no issues that prevent the paper from being properly typeset

You will receive further instructions from the production team, including instructions on how to review your proof when it is ready. Please keep in mind that we are working through a large volume of accepted articles, so please give us a few days to review your paper and let you know the next and final steps.

Lastly, if your institution or institutions have a press office, please let them know about your upcoming paper now to help maximize its impact. If they'll be preparing press materials, please inform our press team within the next 48 hours. Your manuscript will remain under strict press embargo until 2 pm Eastern Time on the date of publication. For more information, please contact onepress@plos.org.

You will receive an invoice from PLOS for your publication fee after your manuscript has reached the completed accept phase. If you receive an email requesting payment before acceptance or for any other service, this may be a phishing scheme. Learn how to identify phishing emails and protect your accounts at https://explore.plos.org/phishing.

If we can help with anything else, please email us at customercare@plos.org.

Thank you for submitting your work to PLOS ONE and supporting open access.

Kind regards,

PLOS ONE Editorial Office Staff

on behalf of

Dr. JONATHAN BAYUO

Academic Editor

PLOS One
